# Diagnosis and Management of Barrett's Esophagus: A Retrospective Study Comparing the Endoscopic Assessment of Early Esophageal Lesions in the Community versus a Specialized Center

**DOI:** 10.1155/2016/5749573

**Published:** 2016-03-29

**Authors:** Erin Rayner-Hartley, Oliver Takach, Cherry Galorport, Robert A. Enns

**Affiliations:** Department of Medicine, Division of Gastroenterology, University of British Columbia, Vancouver, Canada

## Abstract

Specialized endoscopic evaluation for patients with Barrett's esophagus (BE) is well supported; however, no studies have shown that centers with expertise provide better quality care for BE with high-grade dysplasia or early adenocarcinoma. In this study, the investigators aimed to evaluate the management and clinical course for patients treated in a community practice versus a specialized BE center.* Methods*. A retrospective analysis of referrals from the community to our specialized center for evaluation of BE at St Paul's Hospital Division of Gastroenterology between January 2007 and February 2014 was performed. Subjects were patients who were referred for BE and dysplasia and subsequently reevaluated by endoscopy. The pathology and endoscopy reports from the community and our center were reviewed. Inclusion criteria were as follows: being ≥ 19 years old and pathologic diagnosis of BE or dysplasia in the community. Exclusion criteria were as follows: incomplete pathology data or incomplete endoscopy reports from the community physicians.* Results*. A total of 77 patients were reviewed. The staging of 28.9% of patients referred from the community was changed from the initial pathological diagnosis. 18.4% of these patients were upstaged. Using Fischer's exact test, we showed that, in our specialized center, endoscopic impressions correlated significantly with pathology results (*p* < 0.0001).

## 1. Introduction

Barrett's esophagus (BE), a metaplastic transformation of the distal esophagus [1], affects 2% of the population in developed countries [2] and this incidence is rising [3]. Risk factors for BE include gastroesophageal reflux disease (GERD), age of 50 years or older, male sex, race, hiatal hernia, and elevated BMI [4–6].

In BE, the progression from metaplasia to dysplasia and finally adenocarcinoma (AC) has been well established. Dysplasia is subclassified as indeterminate dysplasia, low-grade dysplasia (LGD), or high-grade dysplasia (HGD) [4]. HGD, involving significant distortion of the glandular crypts, is at risk of progression to AC. The classification of AC into intramucosal carcinoma (IMC) versus submucosal carcinoma (SMC) is necessary in order to choose the appropriate management plan (typically endoscopic versus surgical management).

The prevalence of AC in patients with BE and HGD was previously reported to be as high as 40% [7, 8]. More recent studies have shown significantly lower rates of invasive AC, 12.7% and 11.7%, respectively, in patients who underwent esophagectomy for BE and HGD [4, 9]. This overestimate was attributed to the lack of strict pathological definitions of invasive disease. IMC carries a low risk (3-4%) of nodal involvement in contrast to SMC, which carries an 8–33% risk [10].

The management of patients with HGD and AC in BE has evolved in recent years. Surgical esophagectomy was previously the standard of care [8]. The risk of mortality in esophagectomy ranges from 3 to 8% [4]. Shaheen et al. introduced radiofrequency ablation (RFA) as an effective technique for eradicating dysplastic BE in 2009 [11]. Numerous studies have demonstrated better outcomes with endoscopic management of HGD and IMC in BE [4, 12–14]. An endoscopic approach is now recognized as the standard of care for HGD and AC confined to the mucosa [15, 16].

Differentiating IMC from SMC is imperative in deciding whether endoscopic therapy is appropriate for patients with AC in BE. Once visible mucosal abnormalities (VMAs) have been identified, removal of these lesions using endoscopic mucosal resection (EMR) is pivotal for assessment and staging. Correctly identifying and removing IMC while excluding SMC is imperative prior to starting radiofrequency ablation (RFA) [17]. RFA does not provide tissue specimens and thus if submucosal invasion is present, other therapies should be considered. Endoscopic imaging techniques and adherence to biopsy protocols, such as Seattle protocol, have been shown to improve the identification of VMAs that contain HGD or early AC [16]. The use of high definition white light endoscopy (HD-WLE) and narrow band imaging (NBI) increases pathology detection [18, 19]. A recent prospective randomized controlled trial comparing HD-WLE using Seattle protocol with NBI targeted biopsies showed that NBI has similar metaplasia detection rates while requiring fewer biopsies [20].

Specialized endoscopic evaluation for patients with BE is thus well supported, and practice norms of gastroenterologists have been studied. Singh et al. found that only one-third of gastroenterologists use HD-WLE and NBI when assessing BE [21]. Adherence to Seattle protocol for biopsies has been reported between 30 and 50% in the community [22, 23]. These findings suggest that the detection of VMA and early AC may vary depending on the clinical setting. At this time, there are limited studies that have shown that centers with expertise provide different assessments for BE patients with HGD or early AC [24].

A recent Australian prospective cohort study compared detection rates of mucosal lesions and early AC in dysplastic BE patients seen in the community versus a specialized BE unit [25]. 69 patients were referred and reassessed with HD-WLE, NBI, Seattle protocol biopsies, and EMR when appropriate. They found a 56% increased cancer detection rate and suggested that patients with dysplastic BE be considered for referral to a specialized unit.

There is a risk that mucosal lesions harboring dysplasia and cancer may be missed on surveillance endoscopy. We retrospectively compared the endoscopic impression and pathology report in patients with esophageal abnormalities (ranging from intestinal metaplasia and dysplasia to carcinoma) assessed in the community and our specialized BE center. We aimed to compare the overall change in endoscopic impression, which could influence the physician's biopsy plan on initial view. We also aimed to compare the overall change in pathology leading to final diagnosis and patient management.

## 2. Methods

### 2.1. Referrals

We performed a retrospective analysis of referrals from the community to our center for evaluation between 01/07 and 02/14. Physicians specializing in gastroenterology, general surgery, and internal medicine made referrals to our center. Subjects included patients referred for BE, dysplasia, and AC who were subsequently reevaluated by endoscopy. The majority of these patients were referred from outside downtown Vancouver area. The pathology and endoscopy reports from the community and our center were reviewed.

Inclusion criteria included being ≥19 years old and minimum pathologic diagnosis of BE in the community. Exclusion criteria included incomplete pathology data or endoscopy reports from referring physicians.

### 2.2. Assessment

One experienced endoscopist (RE) performed all examinations on the cohort of subjects. All exams were performed using an Olympus Gastroscope (GIF-1TQ160 and/or GIF H180). Biopsies were taken according to Seattle protocol and described using the Prague classification.

### 2.3. Endoscopy Details

Endoscopic impression was retrospectively collected from the initial reports of referring gastroenterologists, surgeons, or internal medicine physicians. This was compared with the endoscopic impression at time of initial evaluation by our specialized gastroenterologist (RE).

### 2.4. Histology Details

One physician (ERH) retrospectively reviewed all referral histopathology reports. More than one specialized pathologist at our institution reviewed all histological findings, unless there was clear evidence of carcinoma on initial evaluation.

## 3. Results

77 patients were referred during this 7-year retrospective review. 62 patients (54 males and 8 females) had sufficient data to be included in the study. Demographic and referral information is summarized in Table 1.

Endoscopic impression was categorized as follows: non-BE, BE, dysplasia, and carcinoma. After assessment in our center, these impressions were changed 56% of the time (35/62 subjects). Overall, 52% of subjects (32/62) were upgraded from the referral impression, 5% (3/62) were downgraded, and 43% (27/62) remained the same. Results are summarized in Table 2.

Referral pathology was categorized as follows: intestinal metaplasia (IM), LGD, HGD, IMC, SMC, and normal. After endoscopic assessment, biopsy, and pathology review in our center, final diagnosis was changed 32% of the time (20/62). 19% (12/62) of subjects were upgraded, 13% (8/62) were downgraded, and 68% (42/62) remained the same as referral. Results are summarized in Table 3.

Subjects with a different pathologic diagnosis after evaluation (32% (20/62)) were assessed. The majority of these subjects (45% (9/20)) were changed from the LGD group. Otherwise, 35% (7/20) were changed from HGD and 10% (2/20) changed from IM and IMC, respectively.

Lastly, outcomes of all patients were assessed. Interestingly, of the 19% (12/62) of upgraded subjects, 58% (7/12) were upstaged to HGD. The majority of these subjects (86% (6/7)) were treated endoscopically with EMR and/or RFA. The outcomes of patients with upgraded pathology are outlined in Table 4.

The correlation between the specialized physician's impression (RE) and pathology at our center was evaluated. Fischer's exact test was used to compare categories of BE and LGD to HGD and AC. We showed that our endoscopic impressions correlated significantly with pathology results (*p* < 0.0001).

## 4. Discussion

This is a single-center retrospective review of patients referred from the community to an advanced endoscopy center for BE, dysplasia, or AC. Esophageal mucosal abnormalities are often subtle and can be challenging to identify. The systematic assessment of the BE segment, including visualization with HD-WLE, NBI, and sampling according to Seattle protocol, increases detection rate [22].

There are several important findings of this study. Firstly, endoscopic impression changed significantly when assessed by an endoscopist experienced in dysplasia. There are several potential explanations for this: imaging equipment (although many communities now have more advanced equipment than tertiary care centers), experience (“one sees only what one is looking for”), or spending more time to carefully analyze the esophagus.

Secondly, pathology and thus final diagnosis were changed in one-third of cases. Interestingly, LGD was the pathological diagnosis which changed most often, representing 45% of changed cases. Possible reasons for this significant number include the following: adherence to biopsy protocols and techniques allowing for improved samples (including the ability to perform EMR at our center), experienced visualization of lesions leading to appropriate sampling, or the specialized assessment by a GI pathologist.

Lastly, our outcomes are compatible with the current standard of care for BE, dysplasia, and IMC, which includes endoscopic assessment and/or treatment. Among subjects with an upgrade in pathological diagnosis, endoscopic treatment was possible in the vast majority of cases (92%, 11/12). This represents the most important group of subjects assessed in this cohort, those at high risk of progression to carcinoma who may have been missed on initial evaluation.

There is minimal data showing that centers with experience in BE provide better care for BE patients with HGD/CA [25]. This study reports a significant benefit in detecting and managing patients with dysplasia. Endoscopists in specialized centers receiving referrals for BE have the advantage of a focused area of expertise, an initial assessment completed by colleagues in the community, a high degree of suspicion, and the luxury of additional time dedicated for evaluation of lesions.

The current expert consensus suggests that patients with BE and at least HGD be managed by gastroenterologists in a center where significant experience and exposure to this disorder exist [24]. Cameron et al. recently showed that patients with at least dysplastic BE were more appropriately investigated in a specialized BE unit. The current study supports this finding.

## 5. Conclusion

Referral and assessment of patients with BE at a specialized center significantly changed initial diagnosis and thus management. This study supports consideration of referral for specialized endoscopic assessment and pathology review. As identified in this review, a small but significant number of patients will likely benefit from early intervention with curative intent.

## Figures and Tables

**Table 1:** Referral details.

	Number
Median age, y	64.5
Gender	
M	8
F	54
Reason for referral	
BE	19
Dysplasia	37
Carcinoma	5
Other	1
Referring physician	
Gastroenterologist	14
Surgeon	37
General internist	11

**Table 2:** Comparison of endoscopic impression at referral and after specialized center evaluation.

	Non-BE	BE	Dysplasia	Carcinoma
*Endoscopic impression*				
At referral	9	49	3	1
After specialized center evaluation	1	33	21	7

*Outcomes of referral endoscopic impression *				
Concordant	0	25	1	1
Downgraded	0	1	2	0
Upgraded	9	23	0	0

**Table 3:** Comparison of most advanced pathology at referral and after specialized center evaluation.

	IM	LGD	HGD	IMC	SMC	Normal
*Pathology*						
At referral	16	13	28	4	1	0
After specialized center evaluation	18	7	29	4	3	1

*Outcomes of referral pathology *						
Concordant	14	4	21	2	1	0
Downgraded	1	2	3	2	0	0
Upgraded	1	7	4	0	0	0

**Table 4:** Outcomes and management of patients with upgraded pathology.

Upgraded pathology	Outcome	Management
IM, *n* = 1	1 upgraded to LGD	1, endoscopic therapy (EMR and/or RFA)

LGD, *n* = 7	7 upgraded to HGD	1, endoscopic surveillance and conservative management (ex PPI)
		6, endoscopic therapy (EMR and/or RFA)

HGD, *n* = 4	2 upgraded to IMC	4, endoscopic therapy (EMR and/or RFA)
	2 upgraded to SMC	
